# *in silico* transcriptome dissection of neocortical excitatory neurogenesis via joint matrix decomposition and transfer learning

**DOI:** 10.1101/2024.02.26.581612

**Published:** 2024-02-28

**Authors:** Shreyash Sonthalia, Guangyan Li, Xoel Mato Blanco, Alex Casella, Jinrui Liu, Genevieve Stein-O’Brien, Brian Caffo, Ricky S. Adkins, Joshua Orvis, Ronna Hertzano, Anup Mahurkar, Jesse Gillis, Jonathan Werner, Shaojie Ma, Nicola Micali, Nenad Sestan, Pasko Rakic, Gabriel Santpere, Seth A. Ament, Carlo Colantuoni

**Affiliations:** 1 Depts. of Neurology, Neuroscience and Biomedical Engineering, Johns Hopkins School of Medicine, Baltimore, MD, USA; 2 Dept. of Biostatistics, Johns Hopkins School of Public Health, Baltimore, MD, USA; 3 Department of Medicine and Life Sciences, Universitat Pompeu Fabra, Barcelona, Catalonia, Spain; 4 Hospital del Mar Research Institute, Parc de Recerca Biomèdica de Barcelona, Catalonia, Spain; 5 University of Maryland, Institute for Genome Sciences, Baltimore, MD, USA; 6 Medical Scientist Training Program, University of Maryland School of Medicine, Baltimore, MD, USA; 7 Neurotology Branch, National Institute on Deafness and Other Communication Disorders, National Institutes of Health, Bethesda, MD, USA; 8 University of Toronto, Toronto, Canada; 9 Yale University, New Haven CT, USA; 10 Maryland Psychiatric Research Center, Department of Psychiatry, University of Maryland School of Medicine, Baltimore, MD, USA; 11 UM-MIND Institute for Neuroscience Discovery, University of Maryland School of Medicine, Baltimore, MD, USA

## Abstract

The rising quality and amount of multi-omic data across biomedical science demands that we build innovative solutions to harness their collective discovery potential. From publicly available repositories, we have assembled and curated a compendium of gene-level transcriptomic data focused on mammalian excitatory neurogenesis in the neocortex. This collection is open for exploration by both computational and cell biologists at nemoanalytics.org, and this report forms a demonstration of its utility. Applying our novel structured joint decomposition approach to mouse, macaque and human data from the collection, we define transcriptome dynamics that are conserved across mammalian excitatory neurogenesis and which map onto the genetics of human brain structure and disease. Leveraging additional data within NeMO Analytics via projection methods, we chart the dynamics of these fundamental molecular elements of neurogenesis across developmental time and space and into postnatal life. Reversing the direction of our investigation, we use transcriptomic data from laminar-specific dissection of adult human neocortex to define molecular signatures specific to excitatory neuronal cell types resident in individual layers of the mature neocortex, and trace their emergence across development. We show that while many lineage defining transcription factors are most highly expressed at early fetal ages, the laminar neuronal identities which they drive take years to decades to reach full maturity. Finally, we interrogated data from stem-cell derived cerebral organoid systems demonstrating that many fundamental elements of *in vivo* development are recapitulated with high-fidelity *in vitro*, while specific transcriptomic programs in neuronal maturation are absent. We propose these analyses as specific applications of the general approach of combining joint decomposition with large curated collections of analysis-ready multi-omics data matrices focused on particular cell and disease contexts. Importantly, these open environments are accessible to, and must be fueled with emerging data by, cell biologists with and without coding expertise.

## Introduction

Expansion of the neocortex has been dramatic in primates, most recently elevating cognitive abilities in the human lineage to extraordinary heights (PMID: 19763105).The neurons of the cortex are born and assembled into networks during prenatal development (PMID: 3291116; PMID: 26796689; PMID: 29170230). Single cell transcriptomic and epigenomic analyses have been used to generate atlases of mammalian neocortical development (PMID: 34616070; PMID: 34390642; PMID: 36509746; PMID: 37758766; PMID: 34163074; PMID: 34321664; PMID: 37824652). Similar maps have been generated of cerebral organoid models (PMID: 30735633; PMID: 30545853; PMID: 36179669), but understanding of the exact elements of development that are recapitulated *in vitro* is lacking. Related datasets are often resident in unlinked databases with incomplete metadata, requiring laborious collection and processing to be interrogated together. To make exploration of multi-omic data more accessible to computational biologists and cell biologists without coding expertise, we have brought grand atlases and individual experiments together in a single analytical environment, including analysis-ready bulk, microdissected, sorted, spatial, and single-cell transcriptomic data spanning cortical development in mouse, primate and human, as well as *in vitro* stem cell models. The Neuroscience Multi-Omic (NeMO) Analytics resource(nemoanalytics.org), where we have assembled these data, can be used to interrogate the expression of individual genes and perform projection analysis to explore complex molecular signatures across the vast collections of datasets.

To more fully leverage these multi-omics data collections, we have applied structured joint decomposition (SJD; doi.org/10.1101/2022.11.07.515489) to define robust shared dynamics across diverse, but biologically linked experiments while avoiding technical artifacts in individual datasets. This allows change within each experiment to be mapped onto relative positions along shared axes of variation, without requiring the geometric alignment of multiple datasets onto a single manifold. Applying SJD to a subset of matrices in the NeMO Analytics collection, we define transcriptomic elements that are conserved across mammalian neurogenesis and map these elements of neocortical neurogenesis onto the genetics of human brain structure and disease. Transfer learning methods implemented within NeMO Analytics (PMID: 32167521; PMID: 31121116; doi.org/10.1101/2022.11.07.515489) enable the tracking of these transcriptomic elements across *in vivo* development and *in vitro* neuronal differentiation models. In a second transfer learning experiment, we generate layer-specific neuronal signatures from adult cortex and monitor their first emergence in early development. We see that layer-specific transcriptomic signatures begin to appear as new-born neurons reach their laminar destination in the developing cortex. Despite their early beginnings, maturation of these laminar identities continues across decades of life. Finally, leveraging the transcriptomic programs we have defined, we show that while cerebral organoids recapitulate general features of cortical neurogenesis, particular elements of pan-neuronal maturation, and upper layer neuronal identities in particular, are not well modeled. We put these analyses forth as examples of the discovery potential that can be achieved through linking vast analysis-ready data resources with matrix decomposition and transfer learning methods, and invite the research community to use and contribute to NeMO Analytics.

## A comprehensive neocortical development multi-omics data exploration environment

**Figure 1:**
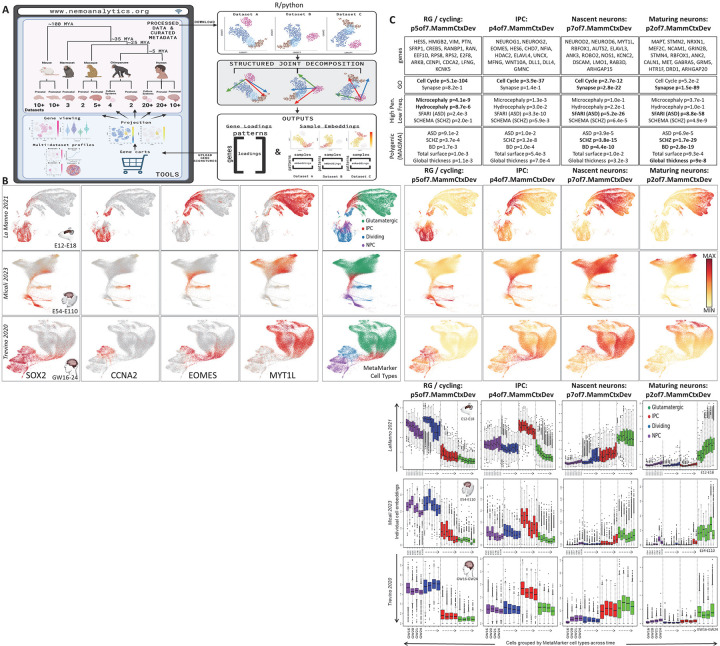
**A]** Schematic of NeMO Analytics data resources employed in conjunction with joint decomposition and transfer learning (projection) approaches. Data can be downloaded from NeMO Analytics and analyzed offline within R or python. Elements learned from this decomposition analysis can then be uploaded back to NeMO Analytics to explore their dynamics across the broad data collection. In this flow, the offline analysis step could be any exploratory technique applied to mutli-omics data matrices that produces gene signatures in the form of simple lists or quantitative loadings, e.g. PCA, WGCNA, or differential expression analysis. **B]** Assembled screen captures from the online NeMO Analytics multi-omic data exploration environment, displaying UMAP representations of scRNA-seq datasets spanning mid-gestational excitatory neocortical neurogenesis in mouse, macaque, and human development colored by the expression of four marker genes (in log2(CPM+1) units). Cell metadata maps at right depict cell labels from the consensus metamarker analysis. **C, upper panels]** Selected genes from the top 0.2% of the gene loadings for 4 of 7 patterns from the jointNMF decomposition of the data from the three species (p7.MammCtxDev). Selected enrichments in each pattern’s gene loadings are also listed (**BOLD** indicates where hits of greatest significance occur for the different phenotypes). All p-values are uncorrected. MAGMA total tests = 7 patterns * 8 MAGMAs = 56 tests. High penetrance, low frequency (geneSetTest) total tests = 12,156. ASD=autism spectrum disorder, SCHZ=schizophrenia, BD=bipolar disorder. See panel D for analysis of gene data underlying the polygenic SCHZ risk enrichment in the maturing neuron pattern p2of7.MammCtxDev. **C, middle panels]** Single-cell embeddings for each of the 4 patterns, displayed as color gradients across UMAP representations. The jointNMF decomposition produces a single gene loading matrix which underlies these sample embeddings, and which we use in downstream functional enrichment analyses to understand the cell biology of these patterns. **C, lower panels]** Boxplots of cell embeddings from each of the 4 NMF patterns we focus on, separated by species, across time and further by metamarker-defined cell type labels. While some patterns appear to distinguish non-overlapping cell types, most span cell type classifications at different levels that are dynamic across cell type and developmental time. Colored lines indicate averages and enrichments of additional gene sets. Panel B and C (middle panels) were created from NeMO Analytics screenshots.

Across the many fields of biomedical research, there is great untapped discovery potential in the high-dimensional multi-omic data currently in the public domain. As new data are published, in addition to data deposition in disparate repositories, researchers often construct custom web portals to provide deep insights into a single novel dataset. Efforts to bring these data together often allow access to data from a large number of studies via the focused exploration of individual datasets one-at-a-time (e.g. cellxgene.cziscience.com, singlecell.broadinstitute.org, and cells.ucsc.edu). With the goal of simultaneously interrogating data from many diverse experiments, at NeMO Analytics (nemoanalytics.org), we have assembled a collection of >100 gene-tabulated transcriptomic and additional multi-omic datasets focused on mammalian excitatory neocortical neurogenesis and related stem cell models (Figure 1A, Table 1, and a full list of dataset at google doc).To expedite analysis, facilitate broad exploration, and maintain reproducible results with original publications, the majority of data in NeMO analytics are formatted as authors originally processed them, most often log transformed and column normalized (e.g. log2(CPM+1) or similar). Using highly curated sample metadata, NeMO Analytics enables the custom assembly and simultaneous visual inspection of many low-dimensional maps of datasets from the collection. Whether derived from a small focused experiment, or the product of “integration” across coordinated studies to create broad molecular atlases, each public data matrix in NeMO Analytics is analyzed and displayed within its own low-dimensional representation, with custom user-created visualizations. This allows assessment of related signals within large numbers of complex, related datasets without the challenges of forcing diverse experimental data into a single unified dimension reduced space. Within NeMO Analytics, researchers can explore measurements of 1] individual genes, e.g. the expression of the DCX gene, marking newborn or immature neurons across a collection of *in vivo* scRNA-seq experiments in mammalian neocortical development (nemoanalytics.org/p?l=NeocortexEvoDevo&g=DCX), or 2] more complex gene signatures, e.g. the summed expression of genes associated with the S or G2M phase of the cell cycle, to distinguish subsets of cycling progenitors across studies in cerebral organoids (nemoanalytics.org/p?p=p&l=NeocortexDevoHsInVitro&c=ed655f33&algo=pca).

The analyses in this report apply joint matrix decomposition approaches to focused collections of transcriptomic data matrices that can be downloaded from NeMO Analytics (Figure 1A, panels at left). The jointly defined dimensions of molecular variation are then uploaded back to NeMO for exploration across its vast processed multi-omic data resources via transfer learning (Figure 1A, panels at right). We propose this as a general strategy for leveraging NeMO Analytics or any analysis-ready data combined with joint decomposition and projection approaches. To demonstrate the breadth and flexibility of visualizations and analyses that are possible within the NeMO Analytics environment, the majority of figures in this report have been assembled entirely from screenshots of the public online resources at nemoanalytics.org.

## Transcriptomic dissection of mid-gestation mammalian neocortical neurogenesis via joint decomposition

To leverage this data collection employing joint decomposition approaches, we assembled scRNA-seq data spanning the excitatory neurogenic trajectory in mid-gestational neocortical development in mouse (PMID: 34321664), macaque (PMID: 37824652) and human (PMID: 34390642). In order to first define a coarse consensus cell labeling across mammalian neocortical development, we used composite expression of “metamarkers” (doi.org/10.1101/2023.03.31.535112), cell type markers that are robust across many studies spanning cortical regions and developmental time (Figure 1B, colored legend). Here we use joint decomposition (SJD, with implementations in R and python via rpy2; doi.org/10.1101/2022.11.07.515489) to define shared dimensions of variation resident within all the three input matrices. SJD can leverage several different matrix decomposition algorithms including, principal component analysis (PCA), independent component analysis, (ICA), and non-negative matrix factorization (NMF) to define shared variation across multi-omics matrices. Here we apply the jointNMF function to decompose transcriptome data across neurogenesis in the three mammals. NMF avoids the orthogonality constraint and conflation that limit PCA, and is therefore well-positioned to identify the many distinct, yet highly correlated signals that are common in multi-omic data. Figure 1C (middle panels) depicts 4 of 7 shared transcriptomic patterns across the single-cell transcriptomic data from mouse, macaque and human that were defined by this first, low resolution joint decomposition (p7.MammCtxDev). The full set of 7 patterns from which these 4 were selected can be explored across these three datasets and additional *in vivo* neocortical development datasets via transfer learning at nemoanalytics.org/p?p=p&l=NeocortexEvoDevo&c=27ca9360&algo=nmf.

These patterns define conserved sequential transcriptomic phases of indirect neocortical neurogenesis spanning radial glial, intermediate progenitors, and distinct neuronal states. The apparent sequential arrangement of neuronal patterns p7of7.MammCtxDev and p2of7.MammCtxDev within the low-dimensional representations of neurogenesis in all three species, along with the individual genes most strongly associated with these two patterns, suggests that p7of7.MammCtxDev is expressed in nascent migrating neurons, while p2of7.MammCtxDev reflects maturation of neurons resident in the cortical plate. Genes with highest loadings in p7of7.MammCtxDev include pro-neural genes NEUROD2 and NEUROD6, while MAPT, GRIN2B, NRXN1, and GABRA5 (genes linked to physical elements of a neuron) are highest in p2of7.MammCtxDev, demonstrating that p7of7.MammCtxDev is a transcriptional program dedicated to becoming a neuron, while p2of7.MammCtxDev represents the neuronal state itself. We will investigate this more deeply in subsequent analyses (Supplementary Table 1 contains the full set of gene loadings across all 7 patterns).

To interrogate these conserved transcriptomic elements of neocortical neurogenesis for association with polygenic risk known to play central roles in neuropsychiatric disease, gene loadings from each pattern were tested for enrichment of gene-level risk estimates from GWAS summary statistics calculated using MAGMA (PMID: 25885710) from 1] recently published studies on neuropsychiatric disease (SCHZ ref: PMID: 35396580, BD ref: PMID: 34002096, ASD ref: PMID: 30804558), and 2] brain MRI structural phenotypes in the UK BioBank (www.ukbiobank.ac.uk). Consistent with many recent observations of their broad polygenic nature (PMID: 31464996; PMID: 37853064; PMID: 31907381), the analysis of genome-wide risk yielded strongest associations for schizophrenia and bipolar disorder with the neuronal transcriptome patterns p7of7.MammCtxDev and p2of7.MammCtxDev (Figure 1C, top panels, in bold). The later neuronal pattern p2of7.MammCtxDev also showed association with brain structure phenotypes. To explore high penetrance, low frequency genetic variation linked to brain structure and disease in these patterns, we conducted enrichment analysis on gene loadings using lists of genes discovered in genome sequencing studies of disease. This revealed that genes linked to diseases which disrupt the gross structure of the brain, including microcephaly (Jin 2020; PMID: 33077954) and hydrocephaly (PMID: 26022163; PMID: 28951247; PMID: 32038172; PMID: 32103185; PMID: 29799801), are associated with p5of7.MammCtxDev that is high in RG and all cycling cells of the developing cortex. Perhaps surprisingly then, p5of7.MammCtxDev is only weakly associated with structural MRI phenotypes assessed using MAGMA and UK BioBank phenotypes (Figure 1C, top panels). We will explore this association of progenitor transcriptional programs with brain structure in more depth in a higher resolution decomposition of these same data in the next section. Genes harboring high penetrance, low frequency variants linked to neuropsychiatric and neurodevelopmental disorders were strongly enriched in the neuronal patterns p7of7.MammCtxDev and p2of7.MammCtxDev, with ASD having particularly strong associations, consistent with many observations indicating that low-frequency, high-penetrance de novo variants play a key role in this disorder (PMID: 32668441; PMID: 35440779). These findings indicate that the distinct genetic architectures underlying these different cortical disorders play out in particular elements of the neurogenic transcriptome (Supplementary Table 2 contains the full list of enrichments across all 7 patterns).

To gain further insight into the cell type specific temporal dynamics of these transcriptomic elements cell embeddings for each pattern in each species were further separated by metamarker cell type and developmental age (Figure 1C, lower panels). The patterns are clearly enriched in individual cell types that are consistent across species, however, there are also significant levels of overlap in signals across multiple cell types. Embeddings within cell types show clear dynamics across age as cells transition through the neurogenic trajectory. Especially clear in the detailed time course of the mouse and macaque data are the descent of progenitor patterns p5of7.MammCtxDev and p4of7.MammCtxDev, and the increase in both the nascent, p7of7.MammCtxDev, and maturing, p2of7.MammCtxDev, neuronal patterns over time. Importantly, these trends are not limited to any one cell type, or even to cell types expressing high levels of the patterns. All classes of neural progenitor have low expression of neuronal patterns p7of7.MammCtxDev and p2of7.MammCtxDev, yet show distinct increases in these signals as development progresses. This has been noted previously by Telley et al 2019 (PMID: 31073041) and Polioudakis 2019 (PMID: 31303374). Similarly, glutamatergic neurons have low levels of progenitor patterns p5of7.MammCtxDev and p4of7.MammCtxDev, and show further decreases over time. The presence of these same signals in laser-microdissected samples from the developing macaque cortex demonstrate that this is not the result of cell-free, or “ambient” RNA contamination in the scRNA-seq data (note VZ in black in NHP LMD from Bakken 2016 in these projections: nemoanalytics.org/p?p=p&l=NeocortexEvoDevo&c=27ca9360&algo=nmf, or the expression of DCX in the same primate data, where DCX expression is low and rising in the germinal zones over time: nemoanalytics.org/p?l=NeocortexEvoDevo&g=DCX).

Notably, the nascent neuron pattern p7of7.MammCtxDev, which is highest in cells called neurons by both the metamarker analysis and the original authors, still shows significant enrichment for cell cycle genes. This suggests a model of neurogenesis where continuous change coexists with the near digital shift from precursor to post mitotic neuron, with neural precursors (NPCs) and intermediate precursor cells (IPCs) being progressively drawn toward the neuronal transcriptome state, and new born neurons continuing to shut down remaining transcriptional elements of their precursor state. This highlights the continuous and overlapping nature of transcriptomic elements resident in individual cells that, especially in development, must be reconciled with the non-overlapping classifications often imposed on single-cells and genes in multi-omic analyses.

There is one clear exception to the observation that the neuronal patterns increase over time in Figure 1C (lower panels): within neurons, while at high levels, the nascent neuron pattern p7of7.MammCtxDev, does not increase further with time. This is in stark contrast to the maturing neuronal pattern p2of7.MammCtxDev, which is high and increases markedly within neurons as time goes on. While we have defined these patterns using only fetal data, this suggests that p7of7.MammCtxDev is transient during neurogenesis, while p2of7.MammCtxDev may be a permanent fixture in the neuronal transcriptome, consistent with the observed extreme enrichment of synaptic genes in p2of7.MammCtxDev. To further explore the function of these transcriptomic signatures, in the next section we will investigate their dynamics across the developmental data collections in NeMO Analytics.

## Exploration of transcriptomic elements of neocortical neurogenesis across the NeMO Analytics data collections via transfer learning

**Figure 2:**
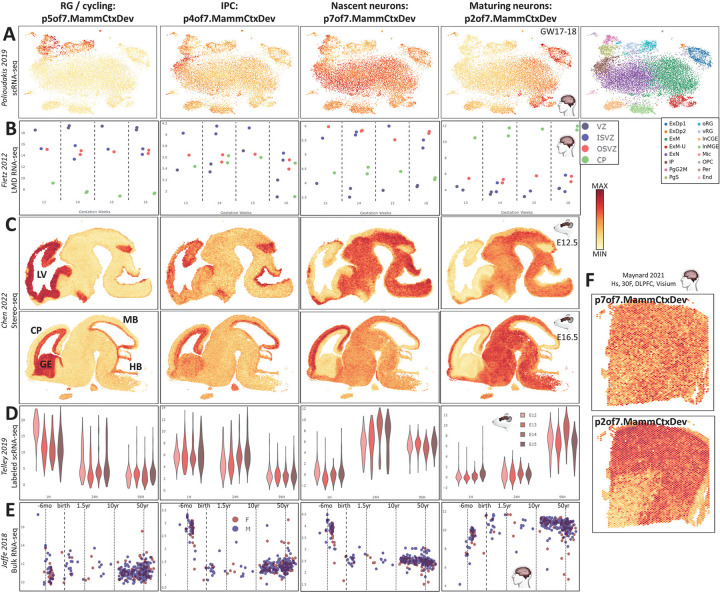
Screen captures of projection of diverse datasets in NeMO Analytics into 4 fundamental transcriptomic elements of neocortical development defined in Figure 1 (p7.MammCtxDev). **A]** tSNE representation of scRNA-seq in fetal human neocortical tissue (GW17–18). **B]** Bulk RNA-seq on LMD samples from human fetal neocortex (GW13–16). **C]** Spatial transcriptomics (Stereo-seq) in the fetal mouse brain. **D]** scRNA-seq of RG cells labeled during their terminal division on the ventricular surface at 0hr and sequenced at 1hr, 24hr, and 96hr (cells labeled at E12-E15, harvested at E12-E19). **E]** bulk RNA-seq of dorsolateral prefrontal cortical tissue across the lifetime. **F]** Spatial transcriptomics (VISIUM) in the adult human dorsolateral prefrontal cortex. LV=lateral ventricle, CP=cortical plate, GE=ganglionic eminence, MB=midbrain, HB=hindbrain. With the exception additional labels, this entire figure was created from NeMO Analytics screenshots.

The gene loadings underlying these transcriptomic patterns were uploaded to NeMO Analytics so that we could leverage its broad multi-omic data collection through projection analysis in order to 1] validate their robustness across measurement technologies, 2] assess their conservation across species and 3] extend our understanding of their temporal and spatial dynamics across development. By running the NMF algorithm or a least squares optimization on new data while using the gene loading matrix from the decomposition of the original datasets, we can estimate embeddings for new cells within the previously defined dimension of transcriptomic variation. These procedures are coded in the projectNMF function in the SJD package (doi.org/10.1101/2022.11.07.515489) and the projectR function in R (PMID: 32167521) and have been implemented within NeMO Analytics for transfer learning experiments. We first validated the neurogenic transcriptome patterns via projection of additional scRNA-seq datasets in the fetal human neocortex. The cellular specificity of these patterns laid out in Figure 1 is consistent across the GW17–18 neocortical data from Polioudakis et. al. 2019 (Figure 2A; PMID: 31303374), with p5of7.MammCtxDev highest in RGs and cycling cells, p4of7.MammCtxDev in IPCs, p7of7.MammCtxDev beginning in IPCs and highest in the newest neurons and p2of7.MammCtxDev in the most mature populations of both deep and upper layer neurons where p7of7.MammCtxDev has begun to descend. Through projection in NeMO Analytics, these findings can be validated and in many additional *in vivo* single-cell human datasets (nemoanalytics.org/p?p=p&l=NeocortexDevoHsInVivo&c=27ca9360&algo=nmf).

Multiple microdissection experiments shed light on the spatial distribution of cells expressing these patterns across the developing cortex (Figure 2B and nemoanalytics.org/p?p=p&l=NeocortexEvoDevo&c=27ca9360&algo=nmf). RNA-seq data from from laser microdissected (LMD) tissue of the developing human neocortex from Fietz et al. (PMID: 22753484) show that the p5of7.MammCtxDev RG and cycling cell signal is highest in the ventricular zone (VZ) where ventricular RGs reside, with only slightly lower levels in the inner and outer subventricular zone (i/oSVZ) where outer RG (oRGs or basal RGs) cells are positioned (PMID: 20154730; PMID: 21127018; PMID: 20436478). The IPC pattern p4of7.MammCtxDev localizes to the iSVZ, where RG cells have delaminated from the ventricular surface epithelium and become IPCs. The nascent neuron p7of7.MammCtxDev is high in both the inner and outer SVZ as the cells’ migration toward the cortical plate begins, with low levels in both the VZ and CP. The maturing neuronal pattern p2of7.MammCtxDev is highest in the CP where new neurons have come to rest at their final destination. This physical progression across the developing cortex is visible in detail in spatial RNA-seq data from Chen et al. (Stereo-seq; PMID: 37442136), where at E16.5 pattern p5of7.MammCtxDev peaks along the ventricles of the telencephalon, p4of7.MammCtxDev moves just off the ventricular surface, expanding radially, and neuronal patterns p7of7.MammCtxDev and p2of7.MammCtxDev continuing this outward progression, with p2of7.MammCtxDev present only in the outer-most layers of the developing CP (Figure 2C). Comparing E12.5 and E16.5, one can see the transition from a developing cortical wall dominated by progenitors to one containing the full laminar organization of these neurogenic states. Looking across regions of the brain reveals the precocious neurogenesis present in more caudal regions of the brain, where p7of7.MammCtxDev peaks at E12.5 and p2of7.MammCtxDev at E16.5.

The Telley et. al. single-cell study (PMID: 31073041) sequenced only cells undergoing their terminal neurogenic division at the ventricular surface at time=0 . Projection of our transcriptomic patterns into this data with precisely birth-dated cells revealed that the conserved p5of7.MammCtxDev RG pattern is highly expressed in ventricular radial glial cells just 1hr after their last division and is reduced quickly thereafter, with very few cells at 24hr expressing high levels of p5of7.MammCtxDev (Figure 2D). Additionally, as observed in the mouse and macaque data in Figure 1C, lower panels, p5of7.MammCtxDev levels also descend in RG (1hr) cells across developmental (pup) ages E12–15. Within cells 1hr after division, the IPC pattern p4of7.MammCtxDev is highest in the subset of cells where p5of7.MammCtxDev is the lowest (nemoanalytics.org/p?l=NeocortexDevoMmInVivo&c=27ca9360&algo=nmf), and is maintained in many cells for 24hr, but is at lowest levels in all cells by 96hr (Figure 2D). The nascent neuronal pattern p7of7.MammCtxDev is not expressed at 1hr and is at highest levels by 24hr after division. Hence, in the mouse, the transcriptomic transition from vRG (high p5of7.MammCtxDev) through IPC (high p4of7.MammCtxDev), to new born neuron (high p7of7.MammCtxDev) takes approximately 1 day. By three days later, at 96hr, p7of7.MammCtxDev is descending and the new neurons are maturing with high levels of pattern 2.

Throughout the *in utero* data sets in Figure 2 we observe the progressive switch first from progenitor pattern p5of7.MammCtxDev to p4of7.MammCtxDev, and then from neuronal pattern p7of7.MammCtxDev to p2of7.MammCtxDev at the most advanced fetal maturity states. This interplay between the nascent and maturing neuronal transcriptomic signatures can be further understood by examining their expression across the lifespan in bulk RNA-seq in human cortex from Jaffe et al. (PMID: 30050107, Figure 2 E). Patterns p5of7.MammCtxDev, p4of7.MammCtxDev, and p7of7.MammCtxDev are all rapidly descending in succession just prior to birth, with all these patterns reaching lifetime minima by birth (Figure 2E). In stark contrast, p2of7.MammCtxDev is steeply increasing over this same prenatal period, arriving at its lifetime maximum at birth, then descending gradually throughout postnatal life and accelerating its descent after 50 years of life, a phenomenon we previously observed in bulk cortical tissue analysis (PMID: 22031444). These effects can also be seen in LMD-coupled microarray data from macaque cortex across pre- and post-natal development from Bakken et al. (PMID: 27409810; nemoanalytics.org/p?p=p&l=NeocortexEvoDevo&c=27ca9360&algo=nmf). This indicates that while arising within just days after a neuron’s birth, p2of7.MammCtxDev becomes a lifelong element of the mature neuronal transcriptome, while p7of7.MammCtxDev is a transient program expressed only at the transition to become a neuron. This is supported by spatial transcriptomic data from the adult human cortex, where p7of7.MammCtxDev shows no specific expression pattern while p2of7.MammCtxDev is high across the entirety of the cortical wall and low in white matter (Figure 2F).

## Higher resolution dissection of the conserved neurogenic transcriptome in neocortex

**Figure 3:**
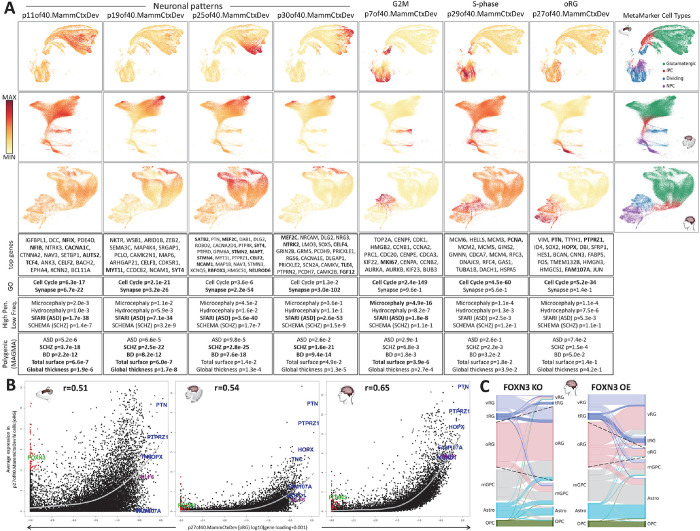
Higher resolution decomposition of the developing neocortical transcriptome (p40.MammCtxDev). **A]** 7 of the 40 patterns defined by jointNMF in the three datasets. **B]** Scatter plots of p27of40.MammCtxDev gene loadings against average expression in putative oRG cells (defined by levels of p27of40.MammCtxDev) in each species. oRG marker genes are shown in blue. Genes in red have low loadings in p27of40.MammCtxDev, but have high expression in mouse oRG cells that have high levels this oRG pattern - among these, FOXN3 is shown in green. KLF6 is shown in purple. The gray curve is a loess fit of the average expression of genes across the magnitude of gene loadings in this pattern. **C]** Cell type transitions predicted by *in silico* knock-out and over-expression simulations in a CellOracle analysis which integrated scRNA-seq and scATAC-seq data from Trevino 2021 (PMID: 36755098) to construct regulatory networks in the developing neocortex. Dashed lines show the expansion of the oRG cell type in FOXN3 KO and its reduction in FOXN3 OE. Images in panel A were created from NeMO Analytics screenshots.

To explore the level of resolution to which we could increase the dissection of this neocortical data collection, we performed a second jointNMF decomposition of the same 3 scRNA-seq data sets from mid-gestation, and in this case defined 40 dimensions (p40.MammCtxDev). The entire set of 40 patterns can be explored across neocortical development at nemoanalytics.org/p?p=p&l=NeocortexEvoDevo&c=052301cb&algo=nmf. Figure 3 depicts the 7 of these 40 patterns across the 3 mammalian datasets, 4 patterns that appear in differing subsets of neurons and 3 that define transcriptional programs within progenitors. Somewhat paradoxically, gene loadings for neuronal patterns p11of40.MammCtxDev and p19of40.MammCtxDev show strong enrichments for both synaptic genes and genes involved in the cell cycle (Figure 3A). This again highlights the fact that transcriptional programs span cell populations that are often defined as discrete non-overlapping classes, but which in fact share many molecular attributes. Interestingly, in addition to highly significant enrichments for neuropsychiatric diseases, enrichments in the genetics of brain structure are greatest in these patterns that appear to bridge proliferative and post-mitotic states in neurogenesis. Patterns p25of40.MammCtxDev and p30of40.MammCtxDev have much less cell cycle involvement and show even stronger enrichments in synaptic as well as disease associated gene sets (Figure 3A). Individual genes with especially high loadings in these patterns include well-known neuropsychiatric risk genes, including RBFOX1 and MEF2C, many synaptic genes, and layer-defining transcription factors (TFs), including SATB2 and TLE4. Hence, neuronal patterns defined in this higher resolution decomposition show enrichment in genes linked to human brain structure and disease, with hits of similar significance to those found in the low resolution decomposition (Figure 1), but here with greater precision in defining the subpopulations of genes and cells that are involved with these associations.

Also in this higher resolution decomposition, transcriptomic elements of neural progenitors and the cell cycle have been separated in greater detail. Two new patterns which span distinct subpopulations of both RGs and IPCs are shown in Figure 3. Both the ranking of individual marker genes among the patterns’ gene loadings (G2M marker MKI67 ranked #22 in p7of40.MammCtxDev and S-phase marker PCNA ranked #5 in p29of40.MammCtxDev) and enrichment analysis (G2M checkpoint in p7of40.MammCtxDev, p=4.9e-76; DNA replication in p29of40.MammCtxDev, p=2.1e-19) clearly indicate that these patterns are present specifically in the transcriptomes of cells in G2M (p7of40.MammCtxDev) and S-phase (p29of40.MammCtxDev) of the cell cycle. In the previous 7 pattern analysis (p7.MammCtxDev), the RG and cycling cell pattern p5of7.MammCtxDev showed enrichment for genes involved in monogenic microcephaly (p=4.1e-9), but only a modestly significant enrichment for genome-wide association with total cortical surface area (p=0.001). Supplementary Table 3 contains the full set of gene loadings across all 40 patterns and Supplementary Table 4 contains the full set enrichments across all 40 patterns. The G2M pattern p7of40.MammCtxDev is present in a much more limited subpopulation of progenitors than p5of7.MammCtxDev, yet showed an even greater enrichment for both monogenic microcephaly (p=4.9e-16) and total cortical surface area (p=3.8e-6). These results indicate that by elevating the resolution of transcriptome decomposition, it is possible to identify increasingly specific transcriptomic elements within cell subtypes and their association with brain structure and disease.

Further breaking down the neocortical progenitor transcriptome, in this analysis of 40 patterns we observed a pattern that is expressed at high levels in thousands of RG cells of the macaque and human, but only in a small number of RG cells in the mouse (Figure 3A, p27of40.MammCtxDev). Author cell type calls and the ranking of the canonical marker HOPX as well as FAM107A, MOXD1, TNC and the ligand-receptor pair PTN-PTPRZ1 (PMID: 26406371), all in the top 100 genome-wide loadings for this pattern, indicate that these cells are oRG cells. oRGs are a primate and human expanded cell type linked to evolutionary increases in neuron number and cortical surface area expansion and account for 40–75% of dividing cells in the developing human neocortex (PMID: 20154730), while accounting for only <10% in the mouse (PMID: 21478886). In addition to the canonical oRG markers, KLF6, a gene recently implicated in evolutionarily new mechanisms in cholesterol metabolism specifically in human oRG cells (doi.org/10.1101/2023.06.23.546307) is also among the top ranked genes in p27of40.MammCtxDev. Additionally, gene loadings for this oRG signature were enriched in genes involved in cholesterol homeostasis (p=6.2e-12).

To explore the individual genes involved in this transcriptomic signature of oRGs, in Figure 3B we have plotted the loading in p27of40.MammCtxDev of each gene against the average expression of that gene in the mouse, macaque or human cells that have highest levels of this oRG signature. In each of the 3 species, we observed the expected strong positive correlation between the p27of40.MammCtxDev loadings and expression levels in high p27of40.MammCtxDev oRG cells. Consistent with a cell type of evolutionarily increasing cohesiveness, this correlation grows from mouse (r=0.51) to macaque (r=0.54) to human (r=0.65), with the relative expression of canonical markers of human oRG cells increasing along this same evolutionary trajectory (Figure 3B). This suggests a model in which a transcriptional regulatory program that began as a diffuse network in RG cells of the mouse cortex has evolved over time to drive the emergence of a novel *bona fide* progenitor type involved in the expansion of the neocortex in the primate and human lineages.

In the mouse specifically, there are many genes which have exceedingly low loadings in p27of40.MammCtxDev while still having high expression in the cells with the highest p27of40.MammCtxDev signal (Figure 3B, red highlighted genes at low X-axis values). In contrast, in both the macaque and human oRG cells, no genes with low p27of40.MammCtxDev loadings are expressed at high levels in these p27of40.MammCtxDev oRG cells. This is consistent with these genes being part of a transcriptional program that has been shut down in oRG cells since the rodent and primate divergence. (Figure 3B). The transcriptional repressor FOXN3 is one of the genes that is high in mouse oRG cells but not in the macaque or human (Figure 3B) and, its targets are enriched in high p27of40.MammCtxDev gene loadings (p=3.9e-20). Integration of scRNA-seq and scATAC-seq data from the human dataset (PMID: 34390642) was carried out in CellOracle (PMID: 36755098) where it is possible to test the simulated effects of TF perturbations on cell identity *in silico*. We found that FOXN3 KO simulation produced many cell state transitions to the oRG state, while over-expression lead to its near disappearance. This suggests the possibility that high FOXN3 expression in mouse oRG cells destabilizes the possible oRG state, reducing numbers of the mouse oRGs, consistent with their sparsity in the rodent brain. This data and the role of FOXN3 as a transcriptional repressor are consistent with a model in which primate oRG cells, where FOXN3 expression is low, allow the increased expression of FOXN3 targets, resulting in the observed enrichment of these targets in the oRG cells, and the stabilization of the oRG cell state in primate and human neocortex.

## Mapping the developmental emergence of adult neuronal laminar identities in the neocortex

**Figure 4:**
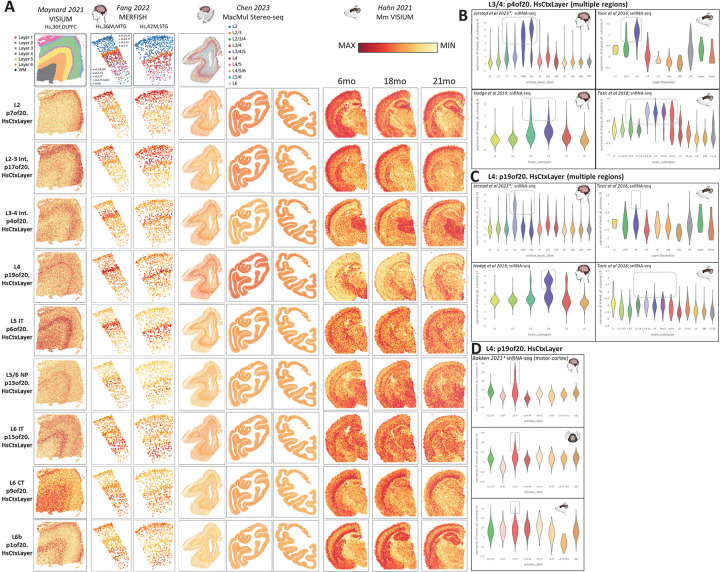
Human adult layer-specific neuronal transcriptome patterns (p20.HsCtxLayer) visualized in additional adult datasets across mammalian species. **A]** Projection of spatial transcriptomic datasets from human, macaque and mouse into the human layer specific transcriptomic patterns. **B]** p4of20.HsCtxLayer is a layer 3/4 transcriptional program conserved from mouse to human. **C]** Across many cortical regions, pattern p19of20.HsCtxLayer captures a primate-specific signature in a subset of L4 neurons that is absent from the mouse neocortex. Data in violin plots has been pooled across brain regions. **D]** In agranular (lacking layer 4) primary motor cortex, p19of20.HsCtxLayer shows high levels in neurons dissected as layer 5 in human and marmoset, but not mouse. * = 1 of 2 studies (5 datasets) used in the joint decomposition that identified this set of p20.HsCtxLayer patterns. CT=corticothalamic, IT=intratelencephalic, NP=near projecting, Int=intermediate. With the exception of additional labels, this entire figure was created from NeMO Analytics screenshots.

To generate a precise quantitative definition of mature laminar-specific neuronal transcriptome identities, we jointly decomposed recent neuronal single-nucleus RNA-seq data from microdissected adult postmortem human cortical tissue from Jorstad 2023 and Bakken 2021 (PMID: 37824655; PMID: 34616062) again using the jointNMF approach in SJD (doi.org/10.1101/2022.11.07.515489). In contrast to marker genes often used to define cellular laminar identities in cortex, here we use 5 snRNA-seq data matrices, each from 1 of 5 distinct donors spanning a total of 8 neocortical regions to define transcriptome-wide signals in the adult neocortex. The 20 patterns from this decomposition (p20.HsCtxLayer) can be explored in detail at nemoanalytics.org/p?p=p&l=0e5c2656&c=1776a2ff&algo=nmf. Supplementary Table 5 contains the full set of gene loadings across all 20 patterns and Supplementary Table 6 contains the full set enrichments across all 20 patterns. Figure 4A shows the projection of spatial transcriptomic data from adult human, macaque and mouse neocortex into 9 of these adult neuronal transcriptome patterns, demonstrating that each pattern has a distinct layer-specific distribution in the adult neocortex of each of the 3 mammalian species studied here.

Pattern p4of20.HsCtxLayer identifies a layer 4 neuronal identity that is conserved in the neocortex of all 3 mammalian species (Fig 4A&B), while pattern p19of20.HsCtxLayer mark a distinct layer 4 transcriptional identity that can be seen in both human and macaque, but not mouse neocortex (Fig 4A&C). Ma et al 2022 (PMID: 36007006) recently observed primate specific expression of FOXP2 (a gene that has been implicated in human language development and neuropsychiatric disease; PMID: 11586359; PMID: 12687690) specifically in excitatory neurons of layer 4. FOXP2 is ranked #144 among the genome-wide loadings for this primate-specific pattern p19of20.HsCtxLayer. While we see high levels of this pattern primarily in layer 4 neurons, the Hodge 2019 snRNA-seq data from adult human cortex also shows small numbers of layer 5 and 3 neurons with elevated p19of20.HsCtxLayer (Figure 4C), also as observed in Ma et al 2022. Chen et al. also recently reported several primate specific layer 4 neuronal cell types in their recent study of spatial transcriptomics in the adult macaque brain (PMID: 37442136). Interrogation of the genes reported in the Chen layer 4 signal in the p19of20.HsCtxLayer gene loadings revealed a significant enrichment of high p19of20.HsCtxLayer loadings in this small group of 11 genes (p=4.5e-6), which includes FOXP2. Hence, the p19of20.HsCtxLayer pattern is likely detecting the same signal as described in Chen 2023, although in that report it was described as several distinct primate specific layer 4 neuronal cell types. To compare with projections of our p19of20.HsCtxLayer pattern, the average expression of the Chen 2023 layer 4 primate-specific gene group can be explored across many datasets with laminar and species specificity at nemoanalytics.org/p?p=p&l=0e5c2656&c=256e16ed&algo=pca.

As observed in Jorstad 2023 (PMID: 37824655), layer 4 transcriptomic identities are present in agranular cortical regions with no histologically identifiable layer 4, including primary motor cortex (Figure 4D, p19of20.HsCtxLayer). This reinforces the notion that although some cortical regions lack large morphologically defined layer 4 pyramidal cells, their shared transcriptomic identities are indeed present in morphologically distinct cells in approximately the same laminar position. Notably, this p19of20.HsCtxLayer transcriptional program is also primate-specific when observed in non-layer 4 neurons (Figure 4D). Similar to the observation of the oRG-related transcriptomic program examined in Figure 3, this suggests that new bona fide cell types may arise through the evolution of novel gene regulatory programs that are initially present in cells with distinct morphologies and functionality.

Three of these cortical patterns also have high expression in specific subcortical regions of the mammalian brain (Figure 4A, Hahn 2021 VISIUM panels: Pattern p1of20.HsCtxLayer is found in both layer 6b and layer 2, in addition to the hippocampus; p4of20.HsCtxLayer is high in layers 3&4 and also in the thalamus; and p7of20.HsCtxLayer is in layer 2, as well as the hippocampus. These regionally defined signals in spatial transcriptomics can also be seen in laser-microdissected samples from the developing and mature macaque brain (PMID: 27409810) via projection (nemoanalytics.org/p?p=p&l=0e5c2656&c=1776a2ff&algo=nmf). These projections clearly demonstrate that multiple human adult laminar neuron transcriptome patterns in the neocortex are shared with neurons in diverse regions of the brain and these shared programs are conserved across mammals, consistent with recent scRNA-seq mapping of neuronal types across the cortex and hippocampus in the mouse brain (PMID: 34004146) where numerous neuronal types were observed to be common across these two brain structures.

**Figure 5:**
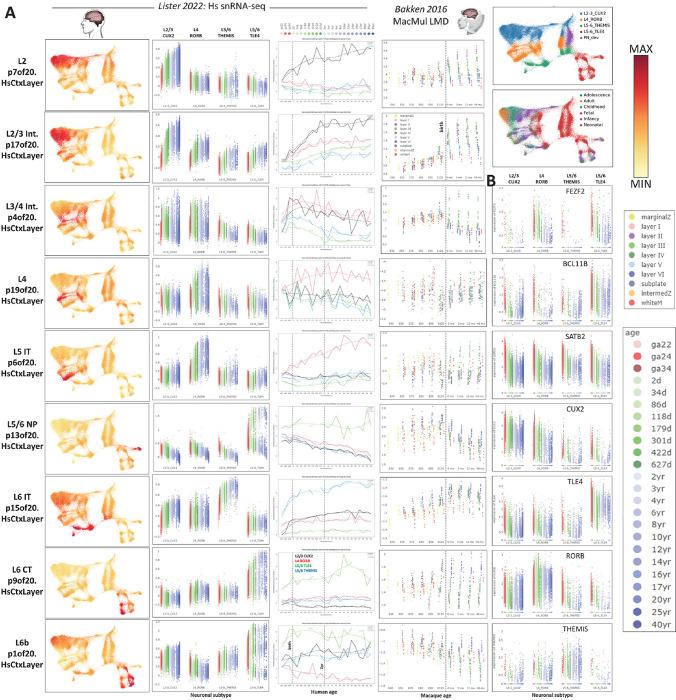
Projection of fetal and postnatal data into adult layer-specific neuronal transcriptome patterns (p20.HsCtxLayer). **A, columns 1–3]** Projection of human snRNA-seq data into the p20.HsCtxLayer patterns, displayed in UMAP dimensions, as strip charts with individual cell embeddings across cell types and ages, and line plots of mean expression levels across age. **A, column 4]** Projection of macaque LMD-coupled microarray data into the p20.HsCtxLayer patterns. **B]** Conventional neuronal marker genes for specific cortical layers. RORB and THEMIS, showing increases in expression in early development, are exceptions to the general pattern of lamina-determining TF expression which peaks at the earliest fetal time points observed here. With the exception of the line plots and additional labels, this entire figure was created from NeMO Analytics screenshots.

To explore the first emergence of these layer-specific neuronal elements of the adult human neocortical transcriptome in development, we examined the expression of these transcriptional programs in snRNA-seq data from neurons in the fetal, postnatal, and mature human neocortex (PMID: 36318921) via projection in NeMO Analytics (Figure 5A and https://nemoanalytics.org/p?p=p&l=9fe511b4&c=1776a2ff&algo=nmf). When visualized on the UMAP of the snRNA-seq data, each of the laminar-specific patterns we derived from adult cortical data appear to occupy a distinct neuronal identity across fetal and postnatal development (Figure 5A, 1^st^ column), indicating that the mature laminar identities emerge early in development. To confirm this, we have depicted these same projections across age as strip charts (showing expression levels in individual cells) and line plots (charting mean expression levels) for each of the neuronal subtypes defined by Lister et al (Figure 5A, 2^nd^ & 3^rd^ columns). Each of the adult layer patterns is more highly expressed in one of the neuronal subtypes distinguished in the developmental data, and these laminar neuronal identities build over developmental time, with lowest levels during fetal ages and increasing in expression over years and decades of postnatal life.

While following this general pattern of building laminar-specific expression over time within a particular neuronal type, two of the three patterns in TLE4+ neurons, p1of20.HsCtxLayer (L6b) & p13of20.HsCtxLayer (L5/6 NP), display particularly unique maturation trajectories. In fetal and early postnatal development, these patterns each rise specifically within distinct sub-populations of TLE4+ neurons and fall in other TLE4+ neurons (Figure 5A, 2^nd^ column). Interestingly, levels of p13of20.HsCtxLayer fall across development and throughout adulthood in all neuron types outside the specific TLE4+ L5 NP subtype in which they are enriched (Figure 5A, 2^nd^ & 3^rd^ columns). Hence, while all layer identities arise from increasing expression levels in a specific neuronal cell type, some also arise via transcriptional repression in other neuronal types. This protracted timeline for the full acquisition of laminar-specific neuronal identities is similar to many elements of human brain development that have acquired longer periods of maturation over evolutionary time (PMID: 30545855; PMID: 37003107; PMID: 34588642).

Understanding of the laminar specificity and developmental trajectories of these mature neuronal patterns can be confirmed and expanded in laser capture microdissection (LMD) coupled analysis (PMID: 27409810, Figure 5A, 4^th^ column). p1of20.HsCtxLayer, which identifies layer 6b in adult human cortex (Figure 4A), is expressed at highest levels in the macaque subplate during *in utero* development, rising postnatally specifically in layer 6. This is consistent with the assertion that layer 6b is the developmental product of the subplate (PMID: 22628460). p9of20.HsCtxLayer emerges in layer 6 across the cortex, and is especially high in layer 6 of a small number of specific dissected samples - these are primary visual cortex, suggesting a regionally specific adaptation of neurons in this layer of V1. p13of20.HsCtxLayer is enriched in deep layers throughout life, but interestingly, decreases over postnatal development across all neuronal subtypes. Peak expression of the layer 4 pattern p4of20.HsCtxLayer is near birth in both human and macaque, except in V1 layer 4c, where it remains at especially elevated levels through postnatal ages.

The prolonged neuronal maturation of laminar identities is in stark contrast to the timing of expression of the individual genes most often employed to distinguish neurons of different neocortical layers, which show highest and most specific expression at the earliest fetal time points (Figure 5B), after which their RNA levels descend as adult laminar identities continue to build. A specific example of this general phenomenon has recently been examined in great detail, where the neuronal lineage-defining TF, FEZF2, was shown to reach peak expression at early fetal ages, just as the deep layer signature it drives is beginning to emerge (doi.org/10.1101/2023.09.12.557406). These observations indicate that while canonical markers of neuronal laminar-specific identities are very effective for the determination of neuronal cell types at very early time points in prenatal development, they are likely poor metrics for layer-specific neuronal maturation, which our transcriptome-wide jointNMF patterns are ideal for. We will use this knowledge to more effectively interrogate neuronal maturity in cerebral organoid models in the next section.

**Figure 6:**
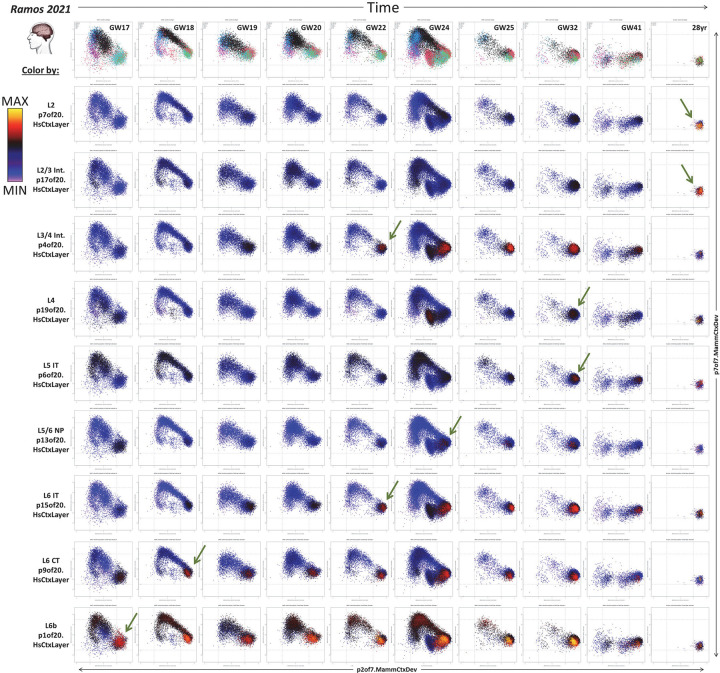
Mapping of snRNA-seq data from pre- and post-natal human neocortex (Ramos 2021, including only cells in the neocortical excitatory neurogenic lineage) into transcriptional dimensions that define neuronal birth and maturation. Each column shows data from a single donor/age. The X-axis in each plot maps the individual cells onto p2of7.MammCtxDev (neuron maturation). The Y-axis maps cells onto p7or7.MammCtxDev (proneural/nascent neurons). The color of points in each row shows the strength of one of the 9 transcriptional programs defined in adult layer-specific neuronal data (p20.HsCtxLayer). Green arrows indicate the 1^st^ age at which each distinct laminar identity emerges clearly in this dataset.

To chart the appearance and maturation of specific cortical laminar identities across their extended developmental trajectories, we combine the broad elements of neuronal maturation captured in the low-resolution decomposition (Figures 1–2; p7.MammCtxDev) with the adult laminar-specific neuronal transcriptional programs (Figures 4&5; p20.HsCtxLayer). Figure 6 shows the transfer of human snRNA-seq data from pre- and post-natal time points (Ramos 2021) into these transcriptional spaces that examine early neuronal maturation in the neocortex. In this series of plots, snRNA-seq data from individual time points in human *in utero* development are mapped onto the two sequential neuronal development dimensions we define in Figures 1&2: p7of7.MammCtxDev (proneural/nascent neurons) and p2of7.MammCtxDev (neuron maturation). These two dimensions, derived from mid-gestation data, precisely describe pan-neuronal birth and early maturation, but do not distinguish between neuronal subtypes. At all ages prior to and including GW24, a clear arc is seen as neurons are born from progenitors at low p7 and low p2of7.MammCtxDev, followed by transient increase in p7of7.MammCtxDev and then a fall in p7of7.MammCtxDev along with p2of7.MammCtxDev induction. After GW24, progenitors and immature neurons begin to disappear, vacating the high p7of7.MammCtxDev and low p2of7.MammCtxDev regions of the plot, coalescing into a single population of low p7of7.MammCtxDev / high p2of7.MammCtxDev cells, where mature laminar identities then emerge (represented as colors in the individual rows of Figure 6). Hence, it appears that mature laminar identities emerge only after new neurons have shut down the nascent/proneural transcriptional program (p7of7.MammCtxDev) and have fully induced the early maturing neuron program (p2of7.MammCtxDev).

The time at which each laminar-specific pattern appears follows the classic inside-out (deep-to-upper) developmental architecture of the neocortex (Figure 6, green arrows). The L3–4 Intermediate pattern p4of20.HsCtxLayer may be an exception to this, emerging earlier than other adjacent neuronal laminar identities, which can also be seen in the Lister 2022 data (Figure 5A). p1of20.HsCtxLayer (subplate & Layer 6b) appears to emerge even before the times sampled in Ramos 2021, and peaks *in utero* between GW22–32 and then descends, likely as the subplate disappears. Layer 2/3 patterns reach peak levels very late (again seen in Lister 2022 in Figure 5).

## Broad in vivo transcriptome dynamics are recapitulated in cerebral organoid models, while specific neuronal maturation trajectories are incomplete

**Figure 7:**
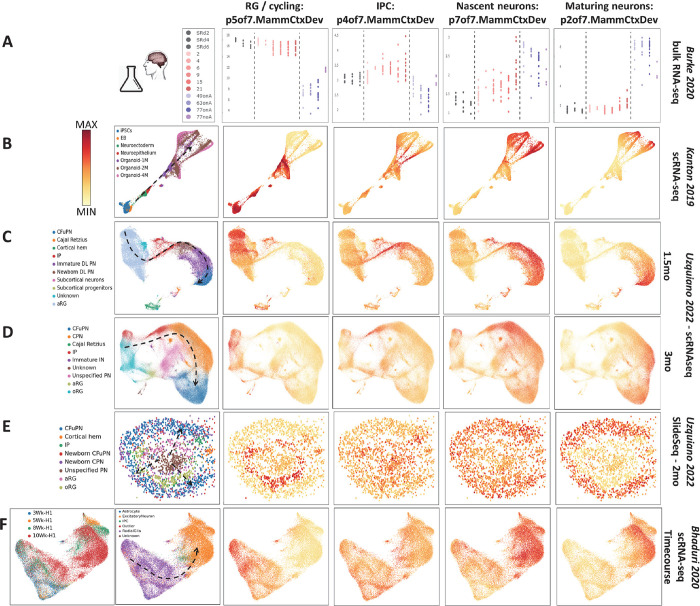
Projection of data from *in vitro* neural differentiation models into the p7.MammCtxDev patterns from Figure 1. **A]** Bulk RNA-seq data from *in vitro* differentiation of 14 hPSC lines from 5 donors. **B]** scRNA-seq from pluripotency through 4 month cerebral organoids. **C&D]** scRNA-seq at single time points in cerebral organoid differentiation. **E]** Spatial transcriptomics in a 2 month cerebral organoid. **F]** scRNA-seq across 3–10 weeks of cerebral organoid differentiation in a single hPSC line using the Xiang 2017 (PMID: 28757360) protocol. Dashed lines indicate approximate neurogenic trajectories in each experiment. This *in vitro* transfer learning experiment examining broad elements of neurogenesis (p7.MammCtxDev) parallels that performed in Figure 2 where *in vivo* data was used. With the exception additional labels, this entire figure was created from NeMO Analytics screenshots.

Two-dimensional neural differentiation and cerebral organoid models have become central tools in modeling development and disease, as well as platforms for the development of novel therapeutics in the CNS. Understanding which elements of development are and which are not modeled with high fidelity is essential to using these models effectively. To address this critical need, we use the transcriptional dimensions that we have defined within *in vivo* neocortical neurogenesis to interrogate human pluripotent stem cell (hPSC) derived models of cortical development *in vitro*. First, we transfer p7of7.MammCtxDev patterns from Figure 1&2 into several *in vitro* datasets, including one 2-dimensional neural differentiation study and three that involved 3-dimensional cerebral organoids. The sequential progression through these broad elements of *in vivo* neocortical neurogenesis defined in the p7of7.MammCtxDev patterns is clear in the bulk RNA-seq from 2-dimensional differentiation, scRNA-seq from organoid models, and spatial transcriptomics data in organoids (Figure 7). The p7of7.MammCtxDev patterns can be explored across these data and a much larger collection of *in vitro* differentiation data at https://nemoanalytics.org/p?p=p&l=NeocortexDevoHsInVitro&c=27ca9360&algo=nmf. While we do not discuss them here in detail, the higher resolution decomposition p40.MammCtxDev patterns from Figure 3 can also be examined across these *in vitro* data at https://nemoanalytics.org/p?p=p&l=NeocortexDevoHsInVitro&c=052301cb&algo=nmf. We have also projected the collection of *in vitro* data into the adult neuron laminar identity patterns p20.HsCtxLayer from Figures 4&5. This assembly of transfer learning experiments showed, as many have observed that deep layer transcriptional identities clearly emerge in neurons *in vitro*, while upper layer identities do not appear systematically. These laminar identity signatures can be explored in stem cell systems at https://nemoanalytics.org/p?p=p&l=NeocortexDevoHsInVitro&c=1776a2ff&algo=nmf.

While *in vivo* neuronal data clearly show reductions in p7of7.MammCtxDev (proneural/nascent neuron) where p2of7.MammCtxDev (maturing neuron) peaks (Figures 2&6), this is not as clear in the *in vitro* models (Figure 7). To examine the neuronal maturation of these systems in more depth, we recreated the transfer learning experiment performed on *in vivo* data in Figure 6, here employing *in vitro* cerebral organoid data (PMID: 31619793; Figure 8A). Early time points contain primarily progenitor states at low p7of7.MammCtxDev and low p2of7.MammCtxDev states. As neurogenesis begins, the elevation of both the proneural nascent neuron pattern p7of7.MammCtxDev and the maturing neuron pattern p2of7.MammCtxDev is clear. Further, as p2of7.MammCtxDev continues to rise, p7of7.MammCtxDev is reduced. These dynamics closely parallel those defined *in vivo* in Figure 6, however, neurons *in vitro* fail to complete this maturational trajectory. At later times as progenitor and early neurons disappear from low p7of7.MammCtxDev and low p2of7.MammCtxDev states, neurons do not continue to reduce p7of7.MammCtxDev and increase p2of7.MammCtxDev, failing to arrive together at a unified low p7of7.MammCtxDev + high p2of7.MammCtxDev state where mature neuron laminar identities emerge as they do *in vivo*. Notably, the strength of mature laminar signals are scattered across cell types and are systematic within low p7of7.MammCtxDev + high p2of7.MammCtxDev expressing neurons only in the subplate/L6b pattern p1of20.HsCtxLayer and the L5/6 NP pattern p13of20.HsCtxLayer, indicating that only a small subset of laminar neuronal identities are progressing beyond the very earliest stages of maturation in the organoids.

**Figure 8:**
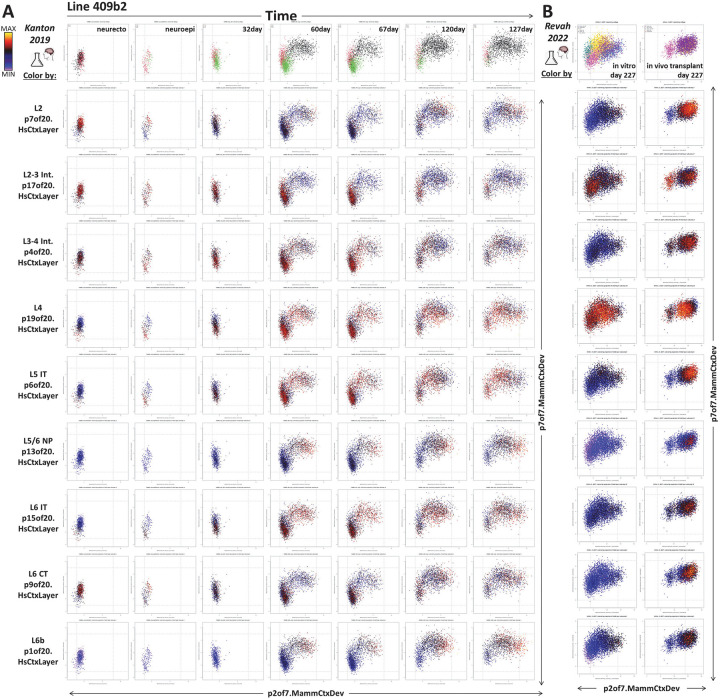
A] Mapping of scRNA-seq data from an in vitro cerebral organoid time course (PMID: 31619793), including only cells in the neocortical excitatory neurogenic lineage) into transcriptional dimensions that define neuronal birth and maturation. Each column shows data from a single donor/age. The X-axis in each plot maps the individual cells onto p2of7.MammCtxDev (neuron maturation). The Y-axis maps cells onto p7or7.MammCtxDev (proneural/nascent neurons). The color of points in each row shows the strength of one of the 9 transcriptional programs defined in adult layer-specific neuronal data (p20.HsCtxLayer). **B]** Transplantation of organoids into the cortex of newborn rat pups elicited significant additional neuronal maturation along specific laminar trajectories over conventionally grown organoids (PMID: 36224417). This *in vitro* transfer learning experiment parallels that performed in Figure 6 that used *in vivo* data.

We have also interrogated the neuronal laminar identities (p20.HsCtxLayer) in data from cerebral organoids generated using diverse protocol modifications, including more or less regionally directed differentiation (PMID: 34616070), longer time courses (PMID: 33619405), and slice preparations (PMID: 30886407). Each of these experiments shows impact on neuronal maturation trajectories, but none fully recapitulate *in vivo* development along the dimensions that we have defined here (nemoanalytics.org/p?p=p&l=NeocortexDevoHsInVitro&c=1776a2ff&algo=nmf). While difficult to assess without a more detailed time course, transplantation of cerebral organoids into the cortex of new born rat pups (PMID: 36224417) induced increased levels of nearly all the adult laminar signatures over conventionally grown organoids, with the possible exception of the primate-specific p19of20.HsCtxLayer pattern (Figure 8B). Continued interrogation of cerebral organoid data as protocols evolve will be necessary to continually asses what elements of *in vivo* development we can effectively explore in these systems.

## Discussion

The NeMO Analytics resources and our decomposition of the transcriptomic dynamics in neocortical neurogenesis can be leveraged to explore development and to design of manipulations of precise cellular mechanisms underlying risk for common complex brain disorders in tractable *in vitro* systems. We invite the research community to explore this collection of public data resources along with the transcriptomic elements of human neurogenesis that we have defined and their projection into *in vitro* stem cell models at nemoanalytics.org, where they are also welcome to upload their own data resources for dissemination and exploration in this neocortical development research environment. It is our hope that the collective exploratory and communication benefits of housing data in this environment will incentivize deposition of emerging neocortical data. We suggest researchers complement their deposition of newly published data in traditional raw data repositories with upload to NeMO Analytics where it will be immediately available to researchers with and without coding experience for exploration alongside the compendium of data on neocortical neurogenesis that we have assembled.

